# A Comprehensive Analysis of *Plasmodium* Circumsporozoite Protein Binding to Hepatocytes

**DOI:** 10.1371/journal.pone.0161607

**Published:** 2016-08-25

**Authors:** Jinghua Zhao, Purnima Bhanot, Junjie Hu, Qian Wang

**Affiliations:** 1 Department of Genetics and Cell Biology, College of Life Sciences, Nankai University, and Tianjin Key Laboratory of Protein Sciences, Tianjin, 300071, China; 2 Department of Microbiology, Biochemistry and Molecular Genetics, Rutgers New Jersey Medical School, Rutgers, The State University of New Jersey, Newark, NJ, 07103, United States of America; 3 National Laboratory of Biomacromolecules, Institute of Biophysics, Chinese Academy of Sciences, Beijing, 100101, China; 4 Department of Immunology, School of Basic Medical Sciences, Tianjin Medical University, and Tianjin Key Laboratory of Cellular and Molecular Immunology, Tianjin, 300070, China; University of Heidelberg Medical School, GERMANY

## Abstract

Circumsporozoite protein (CSP) is the dominant protein on the surface of *Plasmodium* sporozoites and plays a critical role in the invasion by sporozoites of hepatocytes. Contacts between CSP and heparin sulfate proteoglycans (HSPGs) lead to the attachment of sporozoites to hepatocytes and trigger signaling events in the parasite that promote invasion of hepatocytes. The precise sequence elements in CSP that bind HSPGs have not been identified. We performed a systematic *in vitro* analysis to dissect the association between *Plasmodium falciparum* CSP (*Pf*CSP) and hepatocytes. We demonstrate that interactions between *Pf*CSP and heparin or a cultured hepatoma cell line, HepG2, are mediated primarily by a lysine-rich site in the amino terminus of *Pf*CSP. Importantly, the carboxyl terminus of *Pf*CSP facilitates heparin-binding by the amino-terminus but does not interact directly with heparin. These findings provide insights into how CSP recognizes hepatocytes and useful information for further functional studies of CSP.

## Introduction

Species of the genus *Plasmodium* are the causative agents of malaria. Malaria is transmitted through the bite of an infected *Anopheles* mosquito, which injects parasite forms, known as sporozoites, into human hosts. Sporozoites develop in the mosquito midgut inside a structure called an oocyst. After release from the oocyst, sporozoites invade the mosquito salivary glands [1–3]. When an infected mosquito takes a blood meal, sporozoites are deposited into the host’s dermis. The transmitted sporozoites enter the host’s bloodstream, which carries them to the liver. Sporozoites invade hepatocytes and undergo replication within a vacuole to form liver stages. After their release into the bloodstream, the parasites initiate the symptomatic erythrocytic cycle of malaria. Blocking sporozoite infection of the liver decreases both the incidence and severity of the disease, as shown by the use of the recently approved anti-malaria vaccine [4]. This vaccine targets the most abundant protein on the sporozoite surface, the circumsporozoite protein (CSP).

**Fig 1:**
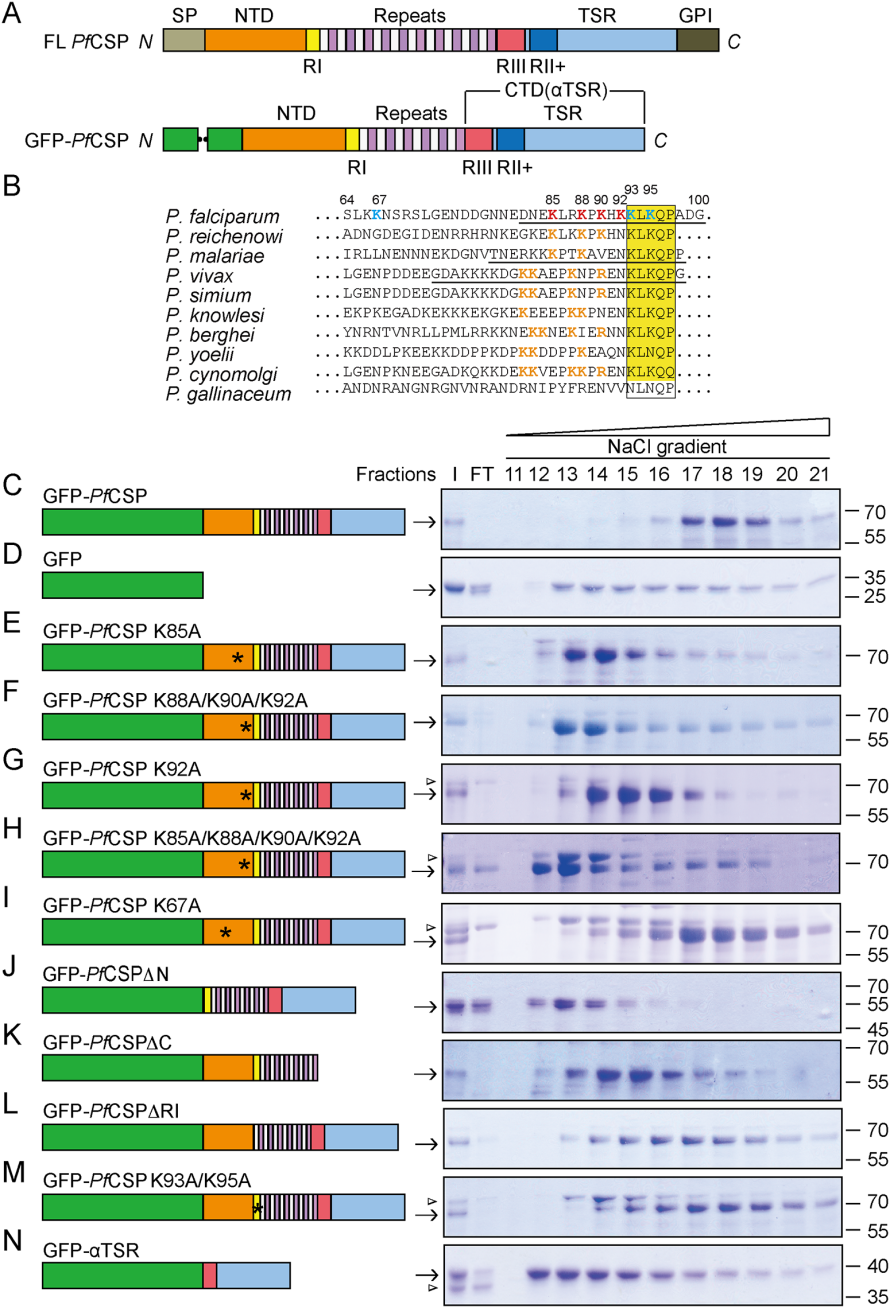
GFP-PfCSP interacts with heparin. **A**, Domain structures of full-length and recombinant *Pf*CSP. The termini are indicated. SP, signal peptide; NTD, N-terminal domain; CTD, C-terminal domain; RI, region I; RII+, region II plus; RIII, region III; GPI, GPI anchor sequence. **B**, Sequence alignment of residues preceding the region I in the NTD of CSP. Residues in *Pf*CSP are numbered, and residues mutated are highlighted in bold. Mutations that affect heparin binding are colored in red and mutation that does not is colored in cyan. Basic residues in other CSPs that are near region I are colored in orange. The region I is highlighted by a yellow box. Peptides that have been tested for heparin binding are underlined. **C**,**D**, Heparin binding of GFP-*Pf*CSP (**C**) and GFP alone (**D**). ~150 μg of purified protein was applied to the heparin column, and samples were analyzed by SDS-PAGE, followed by Coomassie staining. Domain structure of the protein used is shown on the left. I, input; FT, flow-through. **E-N**, as in **C**, but with GFP-tagged CSP mutants. Arrowhead indicates a contaminant. All data were confirmed by at least three independent experiments using three independently purified batches of proteins. Data shown are from a representative experiment.

CSP is present in all *Plasmodium* species. Though variation exists in the amino acid sequence across species, the overall domain structure is well conserved. CSP is embedded in the plasma membrane via a GPI anchor at its C-terminus [5, 6], exposing the protein to the extracellular space. The protein consists of an N-terminal domain (NTD), a region of tandem repeats, and a C-terminal domain that is homologous to the thrombospondin type-1 repeat (TSR) superfamily (Fig 1A). The NTD of CSP lacks predicted secondary structures [7], likely adopting a flexible configuration. Three well-conserved motifs have been identified in CS proteins from different *Plasmodium* species: region I consisting of the peptide KLKQP immediately upstream of the central repeat domain, region III immediately downstream of the repeats, and region II plus in the TSR domain. Recent structural studies have revealed that region III and the TSR fold into a single “αTSR” domain (also referred to as the C-terminal domain, CTD) [8].

CSP plays multiple roles in the parasite life cycle, as it is required for the formation of sporozoites in the mosquito midgut [9, 10], the release of sporozoites from the oocyst [11], invasion of salivary glands [1], attachment of sporozoites to hepatocytes in the liver [12], and sporozoite invasion of hepatocytes [13, 14]. The prevailing model of CSP structure-function suggests that the NTD binds to and masks the C-terminus while sporozoites are in the mosquito salivary glands. In the liver, primary contacts between the N-terminus of CSP and heparin sulfate proteoglycans (HSPGs) on the hepatocyte arrest sporozoites on the hepatocyte surface. Subsequent cleavage of CSP in region I, unmasks the TSR domain. The TSR domain has cell adhesive properties [15], and is thought to mediate the secondary attachment between sporozoites and hepatocytes, thus leading to the invasion of hepatocytes by sporozoites [14]. Sequence elements of CSP that mediate primary and secondary interactions to HSPGs are still unclear.

Full-length CSP is difficult to purify [16, 17] and has not been tested systematically for association with heparin or hepatocytes. Binding assays using peptides or fragments of recombinant CSP suggested that positively charged regions, such as region I and region II plus, are potential binding sites for negatively charged HSPGs [18–20]. However, the physiological relevance of these data is questionable due to probable improper folding or lack of necessary intra-molecular context in CSP peptides or fragments. Attempts have been made to study the CSP structure-function relationship *in vivo* using *Plasmodium berghei* parasites [11, 14]. However, most of these studies did not examine the attachment of sporozoites to hepatocytes and focused on either sporozoite exit from the dermis or sporozoite invasion of hepatocytes. Mutant sporozoites expressing CS protein lacking the entire NTD stick promiscuously to various tissues in the mosquito and dermis [14], suggesting that the TSR domain alone does not facilitate specific interactions between CSP and HSPGs on hepatocytes. These results are consistent with *in vitro* data demonstrating that the purified TSR domain from *Plasmodium falciparum* CSP (*Pf*CSP) does not bind heparin [8]. Furthermore, sporozoites lacking region I alone are not affected in hepatocyte attachment [14]. Taken together, these studies suggest that, during the primary attachment between sporozoites and hepatocytes, the NTD of CSP (excluding region I) contains sequence elements that allow specific interactions between CSP and HSPGs. CSP sequences enriched in basic residues have previously been scanned for heparin binding activity. A region I-containing peptide was found to interact strongly with heparin [20], implicating region I in mediating the contacts between CSP and HSPGs. The sequence upstream of region I contains several basic residues (Fig 1B). The presence of these basic residues raises the possibility that amino acids outside of region I contribute to the binding between CS and HSPGs.

Here, we conduct a systematic analysis of the interaction between CSP and heparin or hepatocytes using GFP-tagged recombinant CS proteins. We found that the NTD of CSP, particularly a lysine cluster upstream of region I, is critical for direct interactions between CSP and HSPGs. Other regions of CSP, including region I and the αTSR domain, may coordinate to present this heparin-binding site indirectly. Our results imply that the lysine cluster is crucial for the specific interaction between CSP and hepatocytes.

## Results

### Interactions between CSP and heparin

To test and visualize the binding of CSP to hepatocytes, we engineered and purified a series of constructs in which the N-terminal signal peptide of *Pf*CSP was replaced by a GFP tag to assist in protein folding (Fig 1A). To further optimize bacterial expression, recombinant *Pf*CSP (residues 19–127 and 256–374) lacked the C-terminal GPI anchor signal and 32 out of 43 repeats. Because CSP is known to recognize heparin, we initially determined the retention of the protein on a heparin column while washing with a salt gradient. As expected, most of the GFP-*Pf*CSP bound to the column (comparing input to flow-through, Fig 1C) and could only be eluted at high salt concentrations (fractions 17–19, Fig 1C). Such binding is unlikely to be due to the GFP tag, as GFP alone mainly flowed through heparin under the same conditions and the retained portion exhibited very weak interactions with the column (Fig 1D). These results suggest that purified recombinant GFP-CSP is capable of binding heparin *in vitro*.

To determine the regions of CSP that could be involved in heparin binding, we examined the NTD for features that are conserved between different *Plasmodium* species in addition to region I. Notably, there is a conserved lysine pattern in CS proteins from different *Plasmodium* species infectious to mammals (Fig 1B), suggesting that these lysines play a conserved functional role. A peptide-based binding assay previously suggested that K85 of *Pf*CSP plays a role in heparin binding [21]. To investigate the role of these lysine residues in heparin binding, we tested the binding of GFP-*Pf*CSP carrying a substitution at K85. GFP-*Pf*CSP K85A eluted from the heparin column at lower salt concentrations compared to GFP-*Pf*CSP (Fig 1E). Substitutions of additional lysines, specifically K88, K90, and K92 individually or in combination, also decreased CSP binding to heparin (Fig 1F, 1G and 1H). The effects of these lysines are specific, as substitution of K67 (K67A) did not significantly decrease heparin binding (Fig 1I). Taken together, the results indicate that the lysine cluster in proximity to region I is most critical for heparin binding.

To investigate whether additional sequence elements of CSP were involved in the interaction with heparin, we truncated either the N-terminus (GFP-*Pf*CSPΔN: deletion of residues 19–92) or C-terminus of *Pf*CSP (GFP-*Pf*CSPΔC: deletion of residues 310–374). GFP-*Pf*CSPΔN was not trapped in the heparin column (Fig 1J), whereas GFP-*Pf*CSPΔC bound to the column but was washed off by relatively low concentrations of salt (fractions 13–16, Fig 1K). These results imply that both termini contribute to interactions with heparin and confirm that the N-terminus is relatively more important.

Several basic regions of CSP have been proposed to engage the negatively charged heparin. These regions include region I, which consists of a conserved “KLKQP” motif (Fig 1B), and region II plus, which consists of a “RKRK”-like motif in the TSR region. Deleting the region I ‘KLKQP’ motif (residues 93–97) from GFP-*Pf*CSP reduced slightly the interaction with heparin, as shown by more protein being eluted in earlier fractions from the column (Fig 1L). The same reduction was observed when the two lysines in region I were substituted with alanine (Fig 1M, K93A/K95A). Thus, mutations in region I have a minor effect on heparin binding, consistent with the normal attachment of sporozoites lacking region I to hepatocytes [14]. Confirming previous results obtained with His-tagged-αTSR [8], GFP-αTSR (residues 310–374, contains region II plus) exhibited marginal binding to the heparin column (Fig 1N). These results, in combination with the reduced affinity of GFP-*Pf*CSPΔC for heparin, suggest that the αTSR domain indirectly influences CSP-HSPG interactions.

**Fig 2:**
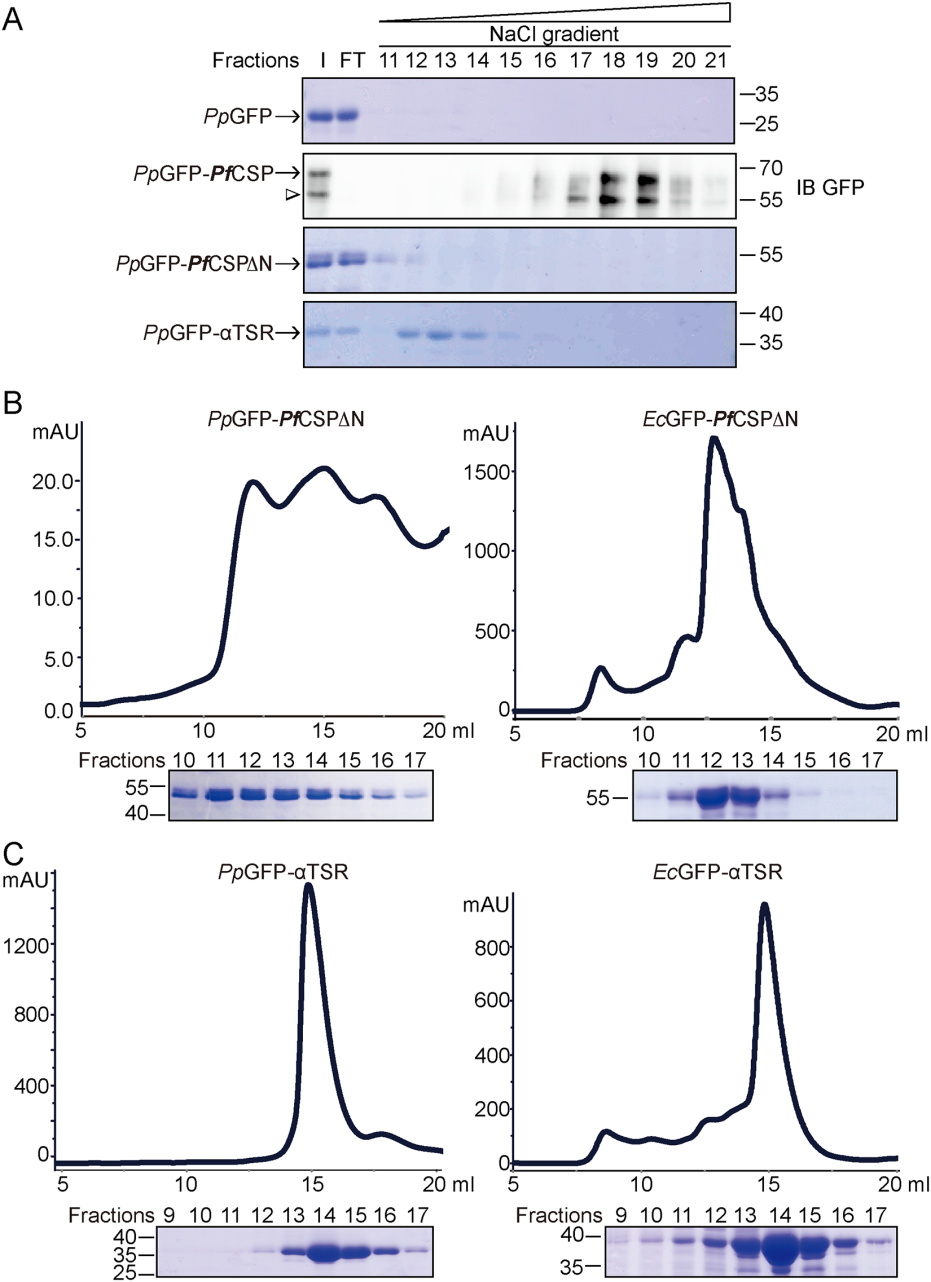
Comparison of recombinant CSP from bacteria and yeast. **A**, Heparin binding of GFP, GFP-*Pf*CSP, GFP-*Pf*CSPΔN, and GFP-αTSR purified from *P*. *pastoris*. Samples for GFP-*Pf*CSP are analyzed by immunoblotting (IB) using antibodies against GFP. All other samples are analyzed by Coomassie staining. A degraded product of GFP-*Pf*CSP is indicated by arrowhead. **B**, Gel filtration analysis of GFP-*Pf*CSPΔN purified from either *E*. *coli* or *P*. *pastoris*. Fractions are analyzed by SDS-PAGE and coomassie staining. **C**, as in **B**, but with GFP-αTSR.

To address the possibility that the lack of binding by GFP-*Pf*CSPΔN is due to poor folding of the CSP produced in the bacterial expression system, we purified GFP, GFP-*Pf*CSP and *Pf*CSP mutant proteins from *P*. *pastoris*, a eukaryotic expression system. We found that GFP-*Pf*CSP purified from *P*. *pastoris* (*Pp*GFP-*Pf*CSP) binds heparin strongly whereas GFP-*Pf*CSPΔN and GFP-αTSR exhibit decreased binding and *Pp*GFP showed no detectable binding (Fig 2A), similar to results obtained from proteins produced in *E*. *coli* (Fig 1B, 1D, 1J and 1N). Though the yield of *Pp*GFP-*Pf*CSP was poor, we recovered sufficient protein for subsequent studies. Gel filtration analysis showed that the GFP-*Pf*CSPΔN and GFP-αTSR from yeast (*Pp*GFP-*Pf*CSPΔN and *Pp*GFP-αTSR, respectively) and *E*. *coli* (*Ec*GFP-*Pf*CSPΔN and *Ec*GFP-αTSR) behave similarly (Fig 2B and 2C). *Pp*GFP-*Pf*CSPΔN and *Ec*GFP-*Pf*CSPΔN proteins have a tendency to oligomerize and elute over the same wide range (Fig 2B). Both *Pp*GFP-αTSR and *Ec*GFP-αTSR behaved normally and were eluted in similar fractions (Fig 2C). Collectively, these results suggest that recombinant CS proteins produced in bacteria and yeast are equivalent in terms of heparin binding.

### Testing interactions between the CSP termini

**Fig 3:**
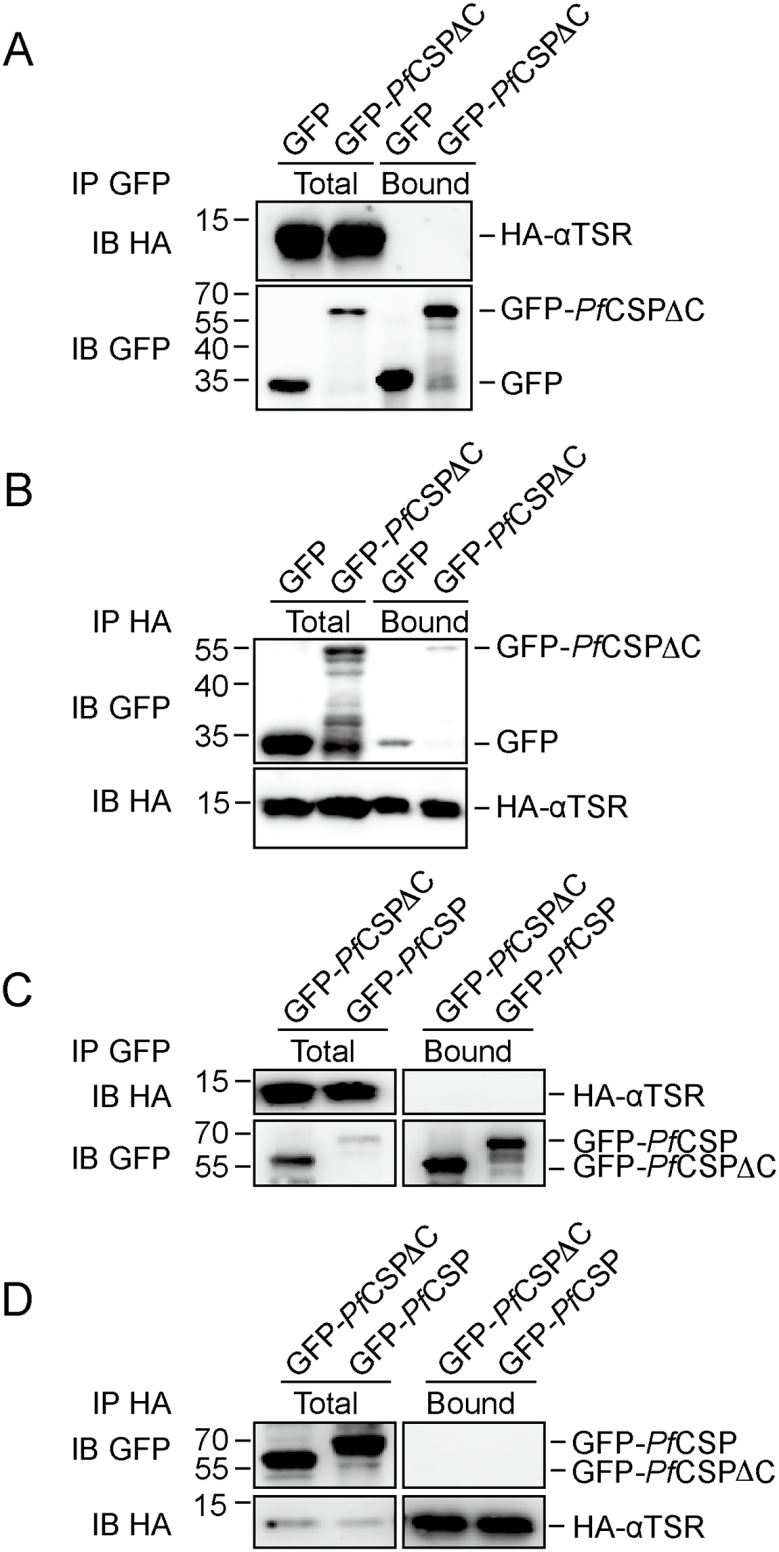
Homotypic interactions between CSP termini. **A**, **B**, Purified GFP-*Pf*CSPΔC or GFP alone was incubated with HA-αTSR. Immunoprecipitation (IP) was performed using anti-GFP (**A**) or anti-HA (**B**) antibodies. The samples were analyzed by SDS-PAGE and immunoblotting (IB) with anti-HA or anti-GFP antibodies. **C**, as in **A**, but with GFP-*Pf*CSP instead of GFP. **D**, as in **B**, but with GFP-*Pf*CSP instead of GFP.

The NTD and CTD of CSP have been proposed to interact with each other prior to proteolysis at region I [14]. To test this interaction, we performed co-immunoprecipitation assays using purified proteins. When GFP-*Pf*CSPΔC was immunoprecipitated using anti-GFP antibodies, there was no detectable co-precipitation of HA-αTSR (Fig 3A). Conversely, when HA-αTSR was precipitated using anti-HA resin, there was only a trace amount of GFP-*Pf*CSPΔC in the precipitate (Fig 3B). However, more GFP alone co-precipitated with HA-αTSR, suggesting that nonspecific interaction between GFP and HA-αTSR account for the trace amount of GFP-*Pf*CSPΔC in the precipitate.

To address the possibilities that incorrect folding of the NTD in *Pf*CSPΔC prevents interaction with HA-αTSR, and that the NTD and CTD interact inter-molecularly, HA-αTSR was incubated with GFP-*Pf*CSP prior to immunoprecipitation with anti-GFP or anti-HA. There was still no detectable co-precipitation (Fig 3C and 3D). These results suggest that the CTD, i.e., the αTSR domain, interacts very little with the NTD of CSP, at least *in vitro*.

### Interactions between CSP and cultured hepatocytes

**Fig 4:**
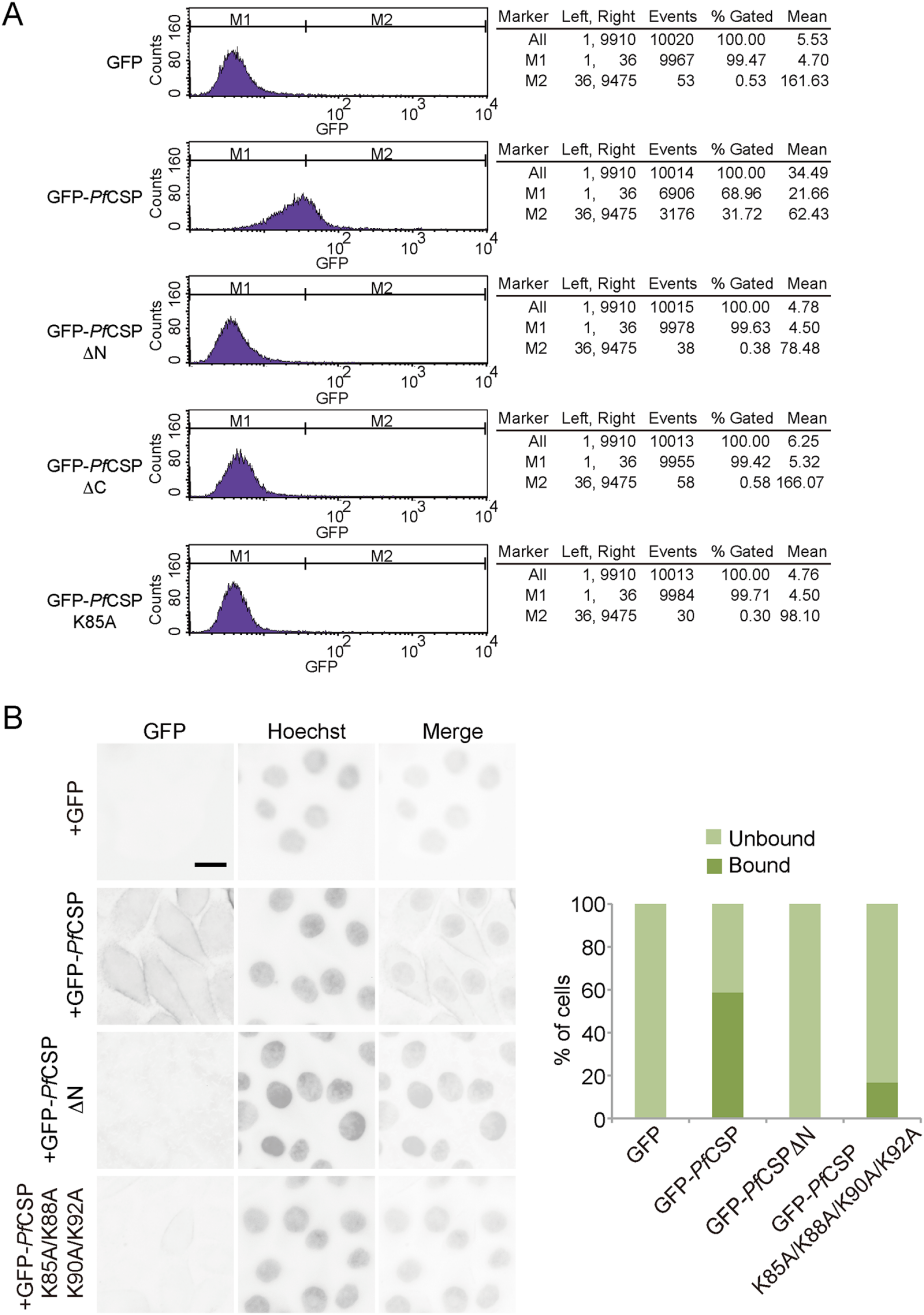
Attachment of CSP to hepatocytes. **A**, Purified GFP-*Pf*CSP or mutant proteins were incubated with HepG2 cells. Binding of the proteins to the cells was quantified by using flow cytometry to detect GFP fluorescence. Analyzed data are shown on the right. The data are representative of at least four repetitions. **B**, Purified GFP-*Pf*CSP or mutant proteins were incubated with HepG2 cells. GFP on the cell surface was detected by live cell imaging. Nuclei were stained with Hoechst 33258 for identification of individual cells. Approximately 300 cells were counted for each sample. The data represent at least three repetitions.

To determine exactly which domain(s) of CSP interacts with liver cells, we incubated different recombinant CS proteins with HepG2 cells and measured their association by flow cytometry using the GFP tag of the recombinant CS proteins for quantification. Incubation of HepG2 cells with GFP-*Pf*CSP, but not GFP alone, significantly increased the GFP signal on the HepG2 cells (Fig 4A). None of the other mutants exhibited detectable differences in HepG2 cell attachment compared to GFP alone (Fig 4A). These results confirmed that the binding of CSP to heparin reflects its binding to liver cells.

To further analyze the activities of the NTD of CSP in a cellular context, we incubated various GFP-tagged CSPs with HepG2 cells and monitored their interactions using live cell imaging. As expected, GFP-*Pf*CSP efficiently decorated the surface of HepG2 cells (Fig 4B). Consistent with flow cytometry analysis, GFP alone or GFP-*Pf*CSPΔN did not bind HepG2 cells (Fig 4B). When the newly identified lysine-rich region of CSP was mutated, interaction with HepG2 were disrupted (Fig 4B). These results confirmed that the NTD of *Pf*CSP, in particular a lysine cluster upstream of region I, is critical for CSP interaction with hepatocytes.

## Discussion

Our data provide important insights into how CSP contributes to the attachment of *Plasmodium* sporozoites to liver cells. We propose that specific lysine residues (K85/K88/K90/K92) upstream of the region I motif in CSP bind to HSPGs on the surface of hepatocytes, likely through electrostatic attraction. One of the lysines, K85 in *Pf*CSP, had been suggested to recognize heparin [21], whereas the other three lysines were not previously identified. The C-terminal α-TSR domain helps stabilize this interaction, though it does not interact directly with either heparin [8] or the NTD of CSP. Region I in the N-terminus is even less important for the heparin binding.

The importance of the identified lysine cluster in mediating CSP interactions with the mammalian liver is supported by domain swapping experiments in which CSP in the rodent-infective parasite *P*. *berghei* was replaced by CSP from the avian-infective species *Plasmodium gallinaceum* (*Pg*CSP) [22]. *Pg*CSP lacks the lysine cluster that we hypothesize is important for targeting sporozoites to the liver. Indeed, *P*. *gallinaceum* sporozoites infect macrophages instead of hepatocytes [23]. Interestingly, the chimeric *P*. *berghei* sporozoites expressing *Pg*CSP were no longer infectious to rodents and failed to invade mosquito salivary glands. Their infectivity to rodents was restored with the re-introduction of the NTD of *P*. *berghei* CSP, implying that CSP sequence elements responsible for mediating liver infection reside in the protein’s N-terminus. These results are consistent with a model in which chimeric sporozoites are not efficiently arrested in the mouse liver due to the lack of lysine residues in *Pg*CSP.

Region I was suggested to contact HSPGs based on experiments utilizing peptides or protein fragments containing region I and the lysine residues identified in the current study [20]. Our data suggest that binding of these peptides to heparin can be explained by the interactions between the lysine residues (K85/K88/K90/K92) and heparin. Consistent with our observation, mutant parasites lacking region I but containing the lysine cluster exhibited normal attachment to Hepa1-6 cells [14]. These results support the notion that region I plays a minor role in the initial binding of CSP on the sporozoite surface to hepatocytes.

Region II plus is also thought to be involved in hepatocyte attachment because of the enrichment of basic residues [24]. Structural studies of the αTSR domain have revealed that region II plus is buried inside the molecule [8], making it less likely that it makes direct contact with other molecules. Indeed, the purified αTSR domain, with intact region II plus, does not bind heparin. Nevertheless, parasites with mutated region II plus exhibit decreased infectivity [11]. One possibility is that the mutation of region II plus distorts the folding of αTSR, which in turn destabilizes the NTD and indirectly affects hepatocyte binding, similar to the GFP-*Pf*CSPΔC mutant tested here. Alternatively, the αTSR domain may bind to molecules other than HSPGs on the surface of hepatocytes to promote secondary attachment to hepatocytes and invasion. Indeed, mutant sporozoites carrying an exposed αTSR domain because of deletion of the preceding NTD of CSP invade even non-liver tissues, such as the dermis [14].

Even though the αTSR domain does not interact with heparin directly, deletion of αTSR affects binding between heparin and the NTD. These results suggest that the CTD can stabilize the NTD. The αTSR domain has also been shown to be accessible to antibodies only when the NTD is cleaved off at region I, suggesting that the CTD is masked by the NTD prior to cleavage [14, 25]. Though a hydrophobic groove was identified in the αTSR domain, suggesting a potential binding pocket [8], the rod-like structure of CSP probed by atomic force microscopy [16] indicated that the terminal association of CSP barely exists or is unstable. Our co-immunoprecipitation assay did not detect an association between purified NTD and CTD. One possibility is that the repeat region, which is largely missing in the recombinant proteins, plays a coordinative role. Alternatively, the interaction is only possible when CSP is properly oriented and assembled on the sporozoite membrane. How the NTD shields the CTD before invasion and how the CTD stabilizes NTD in heparin binding remains to be determined.

We propose that the primary attachment of CSP to HSPGs on the surface of hepatocytes uses the lysine-rich site in the NTD. Following the attachment of sporozoites to hepatocytes, proteolysis at a site in region I exposes the CTD. Finally, the CTD engages the hepatocytes more tightly and promotes invasion by sporozoites [14]. We did not find evidence of an interaction between the CTD and HSPGs, making it likely that the CTD attaches to hepatocytes using molecules other than HSPGs.

Our work also revealed that the attachment of a GFP epitope and reduction in the number of repeats in the central region of CSP greatly facilitates the solubility and purification of CS protein from *E*. *coli*. Previously, CSP expressed recombinantly in *E*. *coli* needed to be refolded to yield soluble proteins [7]. The biophysical properties and heparin-binding abilities of the recombinant modified CSP produced in *E*. *coli* and *P*. *pastoris*, are equivalent. Therefore, the binding analyses in this study are likely to be physiologically relevant. Successful production of large amounts of well-folded CS protein will enable structural studies of CSP and the identification of hepatocyte proteins that interact with the αTSR domain of CSP.

## Materials and Methods

### Plasmid construction

The codon-optimized wild-type CS gene encoding *P*. *falciparum* 3D7 CS (residues 19–127 and 256–374) was fused with a GFP tag at the N-terminus and a His tag at the C-terminus. All point mutations and truncations were generated by overlap PCR using wild-type CS as the template. For *E*. *coli* expression, the wild-type CS and all mutants were inserted into the EcoRI/NotI sites of pET-28a. For *Pichia pastoris* expression, GFP-CS, GFP-CSΔN, and GFP-αTSR were cloned into pPIC9K using EcoRI/NotI sites. All constructs were confirmed by DNA sequencing.

### Protein expression and purification

For *E*. *coli* expression, all constructs were transformed into bacterial strain BL21 (DE3) and cultures grown in Luria-Bertani media at 37°C to an OD_600_ of 0.8. Protein expression was induced by the addition of 0.35 mM IPTG for 24 h at 16°C. Cells were harvested, resuspended in lysis buffer (10 mM Tris pH 8.0, 300 mM NaCl), and lysed by ultrasonication. The lysate was centrifuged at 30,000 rpm for 1 h and the supernatant collected. The protein was isolated on Ni-NTA agarose (Qiagen) and further purified by gel filtration chromatography (Superdex-200; GE Healthcare).

For *P*. *pastoris* expression, SalI linearized plasmids were transformed into *P*. *pastoris* strain GS115 by electroporation. The positive recombinants were then cultured in buffered complex glycerol media followed by protein expression for 48–72 h in buffered complex methanol media (Pichia Expression kit, manual 25–0043; Invitrogen). Culture supernatant supplemented with 0.5 M NaCl and 10 mM imidazole was incubated with Ni-NTA agarose for 1 h at 4°C. After washing with 0.3 M NaCl, 10 mM Tris pH 8.0, and 20 mM imidazole, protein was eluted with 0.15 M NaCl, 10 mM Tris pH 8.0, and 0.5 M imidazole and further purified by gel filtration.

### Heparin binding assay

Purified proteins were diluted with binding buffer (10 mM Tris pH 7.4) and loaded onto a heparin affinity column (Heparin HP 1 ml; GE Healthcare) equilibrated in the same buffer. After washing the column with 5 bed volumes of binding buffer at 0.5 ml/min, the column was developed with a 0–1 M NaCl linear concentration gradient (20 bed volumes) at 0.5 ml/min. Fractions containing target proteins were detected by SDS-PAGE or Western blot.

### Hepatocyte binding assay

The HepG2 hepatoma cell line (ATCC, HB-8065) was maintained at 37°C with 5% CO_2_ in DMEM containing 10% fetal bovine serum. Cells were seeded in a 6-well plate and allowed to adhere for at least 24 h. For FACS analysis, the cells were washed two times with PBS and digested with 0.25% trypsin-EDTA. When the cells detached from the plate, medium was added to terminate the reaction. The cells were harvested and washed one time with PBS and then resuspended with 1 ml PBS, followed by the addition of 20 μg purified protein and incubation at 37°C for 1 h. Unbound protein was removed by washing three times. Finally, cells were resuspended in 500 μl PBS and GFP fluorescence measured by flow cytometry. For microscopy analysis, the cells were incubated with 160–200 μg purified protein at 37°C for 1 h, washed with pre-warmed media and stained with Hoechst at 10 μg/ml. Washed cells were then analyzed live in PBS using Zeiss Observer Z1 with a 40× objective lens.

### GFP pull-down and HA pull-down

For GFP pull-down, 2 μg GFP or GFP fusion protein was incubated with 20 μl Anti-GFP mAb-Agarose (MBL) in 200 μl binding buffer (0.3 M NaCl, 10 mM Tris pH 8.0, 0.5% Triton X-100) for 2 h at 4°C, followed by washing the agarose three times to remove unbound protein. Agarose was resuspended in 500 μl binding buffer and 20 μg purified HA-αTSR added to incubate for 2 h at 4°C. After washing the agarose three times, it was incubated with an equal volume SDS sample buffer. Finally, the samples were subjected to SDS-PAGE and Western blot. HA pull-down was the same as GFP pull-down. A total 2 μg HA-αTSR was incubated with 20 μl Anti-HA-Agarose (Sigma) for 2 h at 4°C. After washing the agarose, GFP or GFP fusion protein was incubated with the washed agarose for 2 h at 4°C in 500 μl binding buffer. After washing the agarose three times and adding SDS sample buffer, the samples were evaluated by Western blot.

## References

[1] Kojin BB, Costa-da-Silva AL, Maciel C, Henriques DA, Carvalho DO, Martin K, et al Endogenously-expressed NH2-terminus of circumsporozoite protein interferes with sporozoite invasion of mosquito salivary glands. Malaria journal. 2016;15(1):153 10.1186/s12936-016-1207-8 26964736 PMC4785649

[2] Ghosh AK, Devenport M, Jethwaney D, Kalume DE, Pandey A, Anderson VE, et al Malaria parasite invasion of the mosquito salivary gland requires interaction between the Plasmodium TRAP and the Anopheles saglin proteins. PLoS pathogens. 2009;5(1):e1000265 10.1371/journal.ppat.1000265 19148273 PMC2613030

[3] Kariu T, Yuda M, Yano K, Chinzei Y. MAEBL is essential for malarial sporozoite infection of the mosquito salivary gland. The Journal of experimental medicine. 2002;195(10):1317–23. 12021311 10.1084/jem.20011876 PMC2193753

[4] Agnandji ST, Lell B, Soulanoudjingar SS, Fernandes JF, Abossolo BP, Conzelmann C, et al First results of phase 3 trial of RTS,S/AS01 malaria vaccine in African children. N Engl J Med. 2011;365(20):1863–75. 10.1056/NEJMoa1102287 . 22007715

[5] Wang Q, Fujioka H, Nussenzweig V. Mutational analysis of the GPI-anchor addition sequence from the circumsporozoite protein of Plasmodium. Cellular microbiology. 2005;7(11):1616–26. 10.1111/j.1462-5822.2005.00579.x . 16207248

[6] Swearingen KE, Lindner SE, Shi L, Shears MJ, Harupa A, Hopp CS, et al Interrogating the Plasmodium Sporozoite Surface: Identification of Surface-Exposed Proteins and Demonstration of Glycosylation on CSP and TRAP by Mass Spectrometry-Based Proteomics. PLoS pathogens. 2016;12(4):e1005606 10.1371/journal.ppat.1005606 27128092 PMC4851412

[7] Plassmeyer ML, Reiter K, Shimp RL Jr., Kotova S, Smith PD, Hurt DE, et al Structure of the Plasmodium falciparum circumsporozoite protein, a leading malaria vaccine candidate. The Journal of biological chemistry. 2009;284(39):26951–63. 10.1074/jbc.M109.013706 19633296 PMC2785382

[8] Doud MB, Koksal AC, Mi LZ, Song G, Lu C, Springer TA. Unexpected fold in the circumsporozoite protein target of malaria vaccines. Proceedings of the National Academy of Sciences of the United States of America. 2012;109(20):7817–22. 10.1073/pnas.1205737109 22547819 PMC3356675

[9] Menard R, Sultan AA, Cortes C, Altszuler R, van Dijk MR, Janse CJ, et al Circumsporozoite protein is required for development of malaria sporozoites in mosquitoes. Nature. 1997;385(6614):336–40. 10.1038/385336a0 . 9002517

[10] Thathy V, Fujioka H, Gantt S, Nussenzweig R, Nussenzweig V, Menard R. Levels of circumsporozoite protein in the Plasmodium oocyst determine sporozoite morphology. EMBO J. 2002;21(7):1586–96. 10.1093/emboj/21.7.1586 11927543 PMC125957

[11] Wang Q, Fujioka H, Nussenzweig V. Exit of Plasmodium sporozoites from oocysts is an active process that involves the circumsporozoite protein. PLoS pathogens. 2005;1(1):e9 10.1371/journal.ppat.0010009 16201021 PMC1238744

[12] Pinzon-Ortiz C, Friedman J, Esko J, Sinnis P. The binding of the circumsporozoite protein to cell surface heparan sulfate proteoglycans is required for plasmodium sporozoite attachment to target cells. The Journal of biological chemistry. 2001;276(29):26784–91. 10.1074/jbc.M104038200 11352923 PMC3941197

[13] Tewari R, Spaccapelo R, Bistoni F, Holder AA, Crisanti A. Function of region I and II adhesive motifs of Plasmodium falciparum circumsporozoite protein in sporozoite motility and infectivity. The Journal of biological chemistry. 2002;277(49):47613–8. 10.1074/jbc.M208453200 . 12244064

[14] Coppi A, Natarajan R, Pradel G, Bennett BL, James ER, Roggero MA, et al The malaria circumsporozoite protein has two functional domains, each with distinct roles as sporozoites journey from mosquito to mammalian host. The Journal of experimental medicine. 2011;208(2):341–56. 10.1084/jem.20101488 21262960 PMC3039851

[15] Adams JC, Tucker RP. The thrombospondin type 1 repeat (TSR) superfamily: diverse proteins with related roles in neuronal development. Dev Dyn. 2000;218(2):280–99. 10.1002/(SICI)1097-0177(200006)218:2<280::AID-DVDY4>3.0.CO;2-0 . 10842357

[16] Plassmeyer ML, Reiter K, Shimp RL, Kotova S, Smith PD, Hurt DE, et al Structure of the Plasmodium falciparum Circumsporozoite Protein, a Leading Malaria Vaccine Candidate. Journal of Biological Chemistry. 2009;284(39):26951–63. 10.1074/jbc.M109.013706. WOS:000269969600075. 19633296 PMC2785382

[17] Noe AR, Espinosa D, Li X, Coelho-Dos-Reis JG, Funakoshi R, Giardina S, et al A full-length Plasmodium falciparum recombinant circumsporozoite protein expressed by Pseudomonas fluorescens platform as a malaria vaccine candidate. PLoS One. 2014;9(9):e107764 10.1371/journal.pone.0107764 25247295 PMC4172688

[18] Rathore D, McCutchan TF, Garboczi DN, Toida T, Hernaiz MJ, LeBrun LA, et al Direct measurement of the interactions of glycosaminoglycans and a heparin decasaccharide with the malaria circumsporozoite protein. Biochemistry. 2001;40(38):11518–24. . 11560500 10.1021/bi0105476

[19] Rathore D, Sacci JB, de la Vega P, McCutchan TF. Binding and invasion of liver cells by Plasmodium falciparum sporozoites. Essential involvement of the amino terminus of circumsporozoite protein. The Journal of biological chemistry. 2002;277(9):7092–8. 10.1074/jbc.M106862200 . 11751898

[20] Ancsin JB, Kisilevsky R. A binding site for highly sulfated heparan sulfate is identified in the N terminus of the circumsporozoite protein: significance for malarial sporozoite attachment to hepatocytes. The Journal of biological chemistry. 2004;279(21):21824–32. 10.1074/jbc.M401979200 . 15007056

[21] Rathore D, Nagarkatti R, Jani D, Chattopadhyay R, de la Vega P, Kumar S, et al An immunologically cryptic epitope of Plasmodium falciparum circumsporozoite protein facilitates liver cell recognition and induces protective antibodies that block liver cell invasion. The Journal of biological chemistry. 2005;280(21):20524–9. 10.1074/jbc.M414254200 . 15781464

[22] Aldrich C, Magini A, Emiliani C, Dottorini T, Bistoni F, Crisanti A, et al Roles of the amino terminal region and repeat region of the Plasmodium berghei circumsporozoite protein in parasite infectivity. PLoS One. 2012;7(2):e32524 10.1371/journal.pone.0032524 22393411 PMC3290588

[23] McCutchan TF, Kissinger JC, Touray MG, Rogers MJ, Li J, Sullivan M, et al Comparison of circumsporozoite proteins from avian and mammalian malarias: biological and phylogenetic implications. Proceedings of the National Academy of Sciences of the United States of America. 1996;93(21):11889–94. 8876233 10.1073/pnas.93.21.11889 PMC38154

[24] Sinnis P, Clavijo P, Fenyo D, Chait BT, Cerami C, Nussenzweig V. Structural and functional properties of region II-plus of the malaria circumsporozoite protein. The Journal of experimental medicine. 1994;180(1):297–306. 8006589 10.1084/jem.180.1.297 PMC2191557

[25] Coppi A, Pinzon-Ortiz C, Hutter C, Sinnis P. The Plasmodium circumsporozoite protein is proteolytically processed during cell invasion. The Journal of experimental medicine. 2005;201(1):27–33. 10.1084/jem.20040989 15630135 PMC1995445

